# Agonist Binding and G Protein Coupling in Histamine H_2_ Receptor: A Molecular Dynamics Study

**DOI:** 10.3390/ijms21186693

**Published:** 2020-09-12

**Authors:** Marcus Conrad, Christian A. Söldner, Yinglong Miao, Heinrich Sticht

**Affiliations:** 1 Bioinformatik, Institut für Biochemie, Emil-Fischer-Centrum, Friedrich-Alexander-Universität Erlangen-Nürnberg (FAU), Fahrstraße 17, 91054 Erlangen, Germany; mar.conrad@fau.de (M.C.); christian.soeldner@fau.de (C.A.S.); 2 Department of Computational Biology and Molecular Biosciences, University of Kansas, Lawrence, KS 66047, USA; miao@ku.edu

**Keywords:** receptor–ligand interactions, G protein-coupled receptors (GPCRs), G_s_ protein, ternary complex, molecular dynamics simulations, metadynamics, Gaussian accelerated simulations (GaMD), ligand binding mode, gastric acid related diseases

## Abstract

The histamine H${}_{2}$ receptor (H_2_R) plays an important role in the regulation of gastric acid secretion. Therefore, it is a main drug target for the treatment of gastroesophageal reflux or peptic ulcer disease. However, there is as of yet no 3D-structural information available hampering a mechanistic understanding of H_2_R. Therefore, we created a model of the histamine-H_2_R-G_s_ complex based on the structure of the ternary complex of the $\beta_{2}$-adrenoceptor and investigated the conformational stability of this active GPCR conformation. Since the physiologically relevant motions with respect to ligand binding and conformational changes of GPCRs can only partly be assessed on the timescale of conventional MD (cMD) simulations, we also applied metadynamics and Gaussian accelerated molecular dynamics (GaMD) simulations. A multiple walker metadynamics simulation in combination with cMD was applied for the determination of the histamine binding mode. The preferential binding pose detected is in good agreement with previous data from site directed mutagenesis and provides a basis for rational ligand design. Inspection of the H_2_R-G_s_ interface reveals a network of polar interactions that may contribute to H_2_R coupling selectivity. The cMD and GaMD simulations demonstrate that the active conformation is retained on a μs-timescale in the ternary histamine-H_2_R-G_s_ complex and in a truncated complex that contains only G_s_ helix α5 instead of the entire G protein. In contrast, histamine alone is unable to stabilize the active conformation, which is in line with previous studies of other GPCRs.

## 1. Introduction

Histamine is an important tissue hormone which is primarily detected by human cells via the four histamine receptors H_1_R, H_2_R, H_3_R and H_4_R [1,2]. All of them belong to the class A of G protein-coupled receptors (GPCRs) [3]. Binding of histamine favors the transition from inactive towards active subsets of receptor conformations which allows for the coupling to intracellular binding partners (IBPs) [4,5]. Each histamine receptor couples to a specific subset of IBPs including several types of G proteins and arrestins, which participate in a plethora of different pathways and thus lead to various changes within the behavior of the cell and the surrounding tissue [3]. While the H_1_R is especially relevant for inflammatory and allergic reactions [6], the main role of the H_2_R is regulation of gastrointestinal motility, intestinal and most notably gastric acid secretion [7]. Therefore, H_2_R antagonists are used in the treatment of gastroesophageal reflux or peptic ulcer disease [8,9]. Furthermore, H_2_R is expressed in myocardial cells [10]. Recent studies suggested that H_2_R antagonists may reduce the risk of heart failure (HF) [11]. Patients with HF that were subjected to treatment with H_2_R antagonists were shown to have a less alarming cardiac morphology and in general weaker symptoms [12]. Despite its pharmacologic importance, until now there is no 3D-structural information available for H_2_R hampering a mechanistic understanding and rational design of drugs to modulate H_2_R activity.

We have generated a computational model of the ternary histamine-H_2_R-G_s_ complex and simulated its conformational dynamics. Both ligand binding and conformational changes of GPCRs frequently occur on timescales longer than microseconds [13,14] and can therefore only partly be assessed by conventional molecular dynamics (cMD) simulations. To enhance conformational sampling, we have applied metadynamics and Gaussian accelerated molecular dynamics (GaMD) simulations in this study.

Metadynamics is a method which accelerates sampling along one or more collective variables (CVs) by adding gaussian potentials to the free energy landscape at regular time intervals. These are centered at the evaluated CV values of the respective frame and aim to facilitate the exploration of so far unvisited configurations. This history-dependent potential reduces the probability of resampling the same position in CV space [15,16,17]. By using the multiple walker approach, which introduces several simultaneous simulations with shared potentials, metadynamics gets even more efficient. To date, metadynamics simulations have been successfully used to study GPCR-ligand interactions in a couple of cases including opioid [18,19], vasopressin [20], chemokine [21], or cannabinoid receptors [22].

GaMD works by applying a harmonic boost potential to reduce system energy barriers and accelerate biomolecular simulations by orders of magnitude [23]. The boost potential can be applied to dihedral torsions (dihedral-boost GaMD) or the system total potential energy (total-boost GaMD) or both (dual-boost GaMD). In contrast to metadynamics, GaMD does not require carefully chosen CVs. GaMD can thus be used for simulations of complex biological processes without the need of prospective knowledge of the studied system [23,24]. It also provides unconstrained enhanced sampling. Moreover, because the boost potential exhibits a Gaussian distribution, biomolecular free energy profiles can be properly recovered through cumulant expansion to the second order [23]. GaMD simulations have successfully revealed mechanisms of protein folding and conformational changes [23,25,26,27,28,29], ligand binding [23,26,27,30,31,32,33,34,35], and protein-protein/membrane/nucleic acid interactions [32,36,37,38,39,40].

In the present study, a multiple walker metadynamics simulation was applied to deduce the histamine binding mode in H_2_R, which was subsequently refined by 1 μs cMD simulations according to a previously established strategy [41]. GaMD was applied in addition to assess the role of different binding partners in the conformational stability of the active H_2_R. We compared dynamics of the histamine-H_2_R-G_s_ ternary complex, the binary complex of histamine-H_2_R and the apo H_2_R without any binding partner. In addition, a truncated ternary complex that contains only the C-terminal G_s_-α5 helix instead of the entire heterotrimeric G_s_ protein was simulated to investigate whether the α5 helix can sufficiently stabilize the active state. For the comparison of these four systems, a total of 8 cMD and 14 GaMD simulations (1 μs length each) were performed.

Using the computational strategy described above, we were able to generate a model of the ternary histamine-H_2_R-G_s_ complex and to characterize the H_2_R-histamine and H_2_R-G_s_ interfaces in detail. In addition, simulations of H_2_R in complex with different binding partners provided important insights into structural dynamics of the active H_2_R and its conformational transitions in the absence of intracellular binding partners.

## 2. Results and Discussion

### 2.1. Modeling of the H_2_R in the Active State

**Figure 1:**
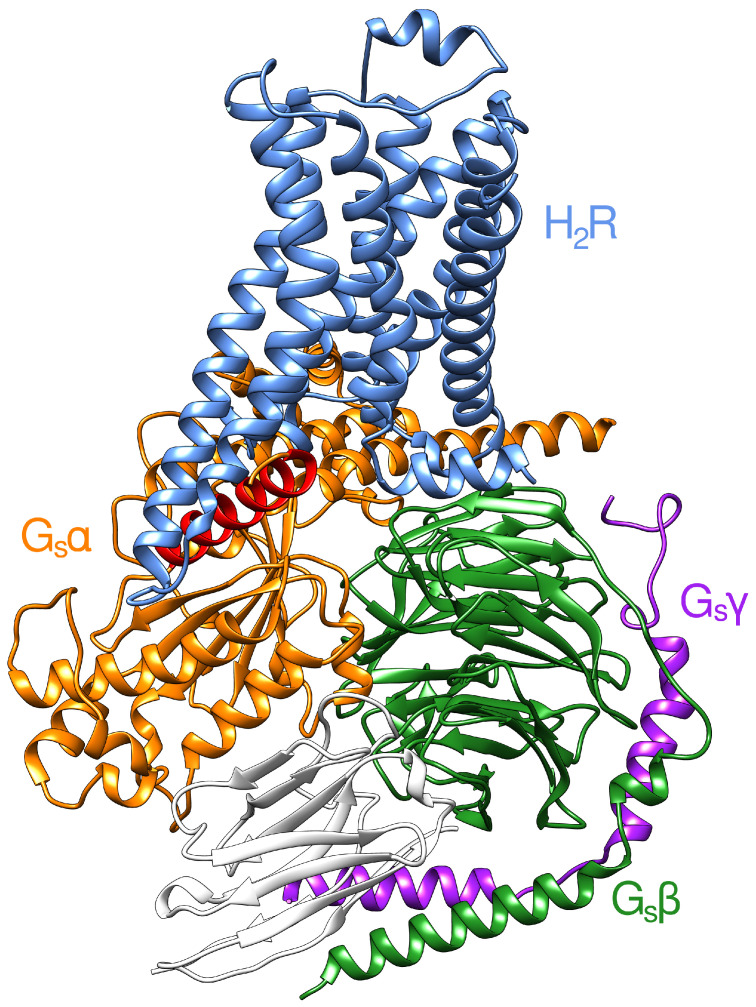
Homology model of the H_2_R-G_s_ protein complex. For clarity, only the backbones are shown in ribbon representation. The H_2_R is colored in light blue, the Gsα subunit is orange with the C-terminal α5 helix highlighted in red. The Gsβ and Gsγ subunits are shown in green and violet, respectively. The camelid antibody fragment which was kept for stabilization is colored in white.

To obtain a model of H_2_R in the active state, we included both an agonist (histamine) in the orthosteric pocket and a G protein at the cytosolic side. H_2_R efficiently couples to G_s_, but may also bind other types of G proteins [7,42]. Therefore, we used the crystal structure of the human β_2_ adrenoceptor in complex with G_s_ (PDB code 3SN6 [43]) as template to generate an H_2_R model in the active state (see Methods section for details). The resulting model (Figure 1) still lacks the agonist histamine, for which the binding mode was determined in the second step by molecular dynamics simulations.

**Figure 2:**
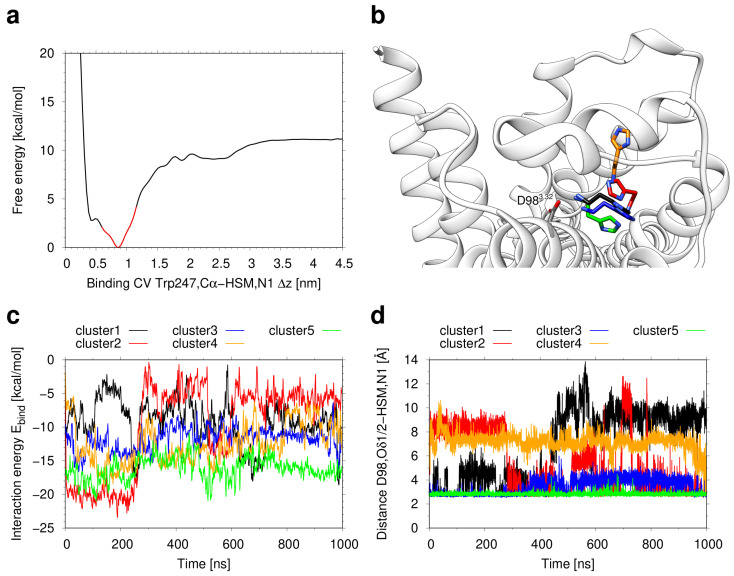
Deduction of the histamine binding mode. (**a**) Free energy landscape as a function of the binding collective variable, i.e., the *z* component of the distance between the Cα atom of W247^6.48^ and the ammonium nitrogen atom of histamine (HSM). The red section of the graph within 3 Å of the global minimum highlights the subset of the structures which was extracted for clustering. (**b**) Representative structures for the five clusters obtained from the frames around the free energy minimum. The backbone of the H_2_ receptor is shown as white ribbons. The conformations of histamine are displayed as sticks. The carbon atoms are colored in black (cluster1), red (cluster2), blue (cluster3), orange (cluster4), and green (cluster5). (**c**,**d**) Conventional refinement MD simulations of the five cluster representatives. (**c**) MM/GBSA interaction energy between histamine and the H_2_ receptor as a function of simulation time. (**d**) Distance of the salt bridge between the histamine ammonium nitrogen atom and the Oδ1/2 atoms of D98^3.32^ as a function of simulation time. For every frame, the shorter one of both distances is shown.

The simulation of histamine binding to the H_2_R was done using the previously established protocol from Söldner et al. [41] that combines multiple walker metadynamics simulations with a ligand clustering protocol and refinement by cMD. The free energy landscape derived from the respective metadynamics simulation is shown in Figure 2a. The shape of this curve is similar to those observed in previous ligand binding simulations [41,44] and indicates an energy minimum within the orthosteric pocket of the receptor. According to the strategy from Söldner [41] structures within 3 Å of the global minimum (red segment in Figure 2a) were grouped into five clusters. The representative structures from these clusters were all located in the orthosteric pocket but exhibited certain differences in their orientation and interactions in the pocket (Figure 2b).

To assess the conformational stability and refine ligand binding mode of the H_2_R, subsequent 1-μs MD simulations were performed for the representative structures from all five clusters. According to the binding energy (Figure 2c), cluster5 exhibits the tightest interactions and the lowest fluctuations over simulation time. The high conformational stability of cluster5 also becomes apparent from inspection of the distance between the histamine ammonium group and the carboxyl group of D98^3.32^, which represents a key ionic anchor in aminergic GPCRs [45]. Again, cluster5 exhibits the tightest interaction and smallest fluctuations indicating the highest stability of this binding mode (Figure 2d). Based on the observations above, we considered cluster5 as the most favorable interaction mode and inspected the histamine-receptor interactions in more detail.

**Figure 3:**
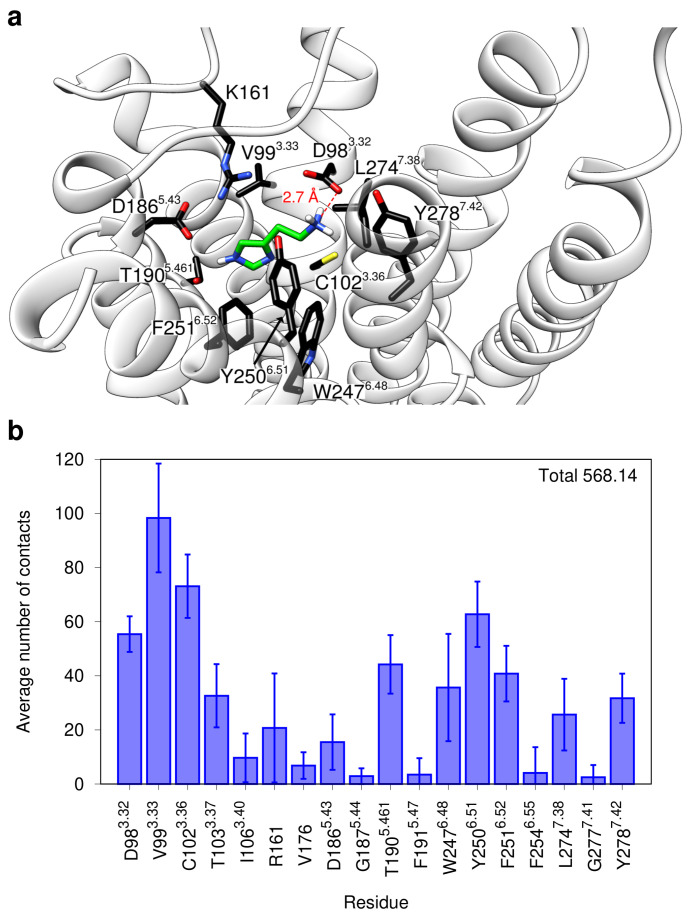
Binding mode of histamine within the H_2_R. (**a**) Binding position of histamine within the orthosteric pocket of the H_2_R as deduced by the refinement MD simulation of cluster5. The receptor backbone is drawn as white ribbons. Histamine is displayed as sticks with the carbon atoms colored in green. All residues within 3 Å of the ligand are shown as sticks with black carbon atoms. (**b**) Number of contacts between histamine and individual H_2_R residues. The columns show per-frame averages for the refinement simulation of cluster5 whereas the error bars indicate the respective standard deviations. Only residues with an average of at least 1 contact per frame were taken into account.

In addition to the salt bridge between the histamine ammonium group and D98^3.32^, polar interactions exist between one imidazole nitrogen and the sidechains of D186^5.54^ and T190^5.461^ (Figure 3a). This finding is in good agreement with data from site-directed mutagenesis: Mutation of the acidic residues (D98N or D186A) abolished both [^3^H]-methyltiotidine binding and histamine-stimulated increases in cAMP content [46]. T190A or T190C mutations retained the ability to bind [^3^H]-methyltiotidine, although with significantly reduced affinity [46]. The remaining interactions between histamine and H_2_R are mostly hydrophobic. Residues that form a large number of contacts over the simulation time include V99^3.33^, C102^3.36^, W247^6.48^, Y250^6.51^, F251^6.52^, and Y278^7.42^ (Figure 3a,b).

### 2.2. Interface between the Receptor and the G_s_ Protein

**Figure 4:**
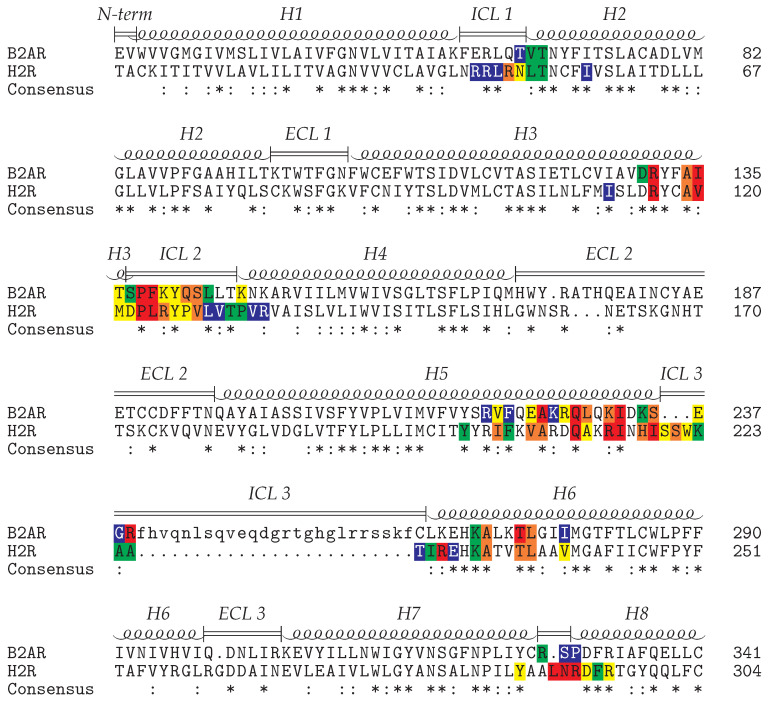
Sequence alignment of the histamine H_2_ receptor (H2R) and the $\beta_{2}$ adrenoceptor (B2AR). The sequences are shaded according to the number of contacts (red > orange > yellow > green > blue) that the respective residues formed with the G_s_ protein during the simulations (H_2_ receptor: H-G_s_-cMD1, H-G_s_-cMD2) or in the crystal structure ($\beta_{2}$ adrenoceptor: PDB code 3SN6). The approximate length of the structural elements is indicated above the alignment. The ICL3 residues in lower case letters are not resolved in the $\beta_{2}$-AR crystal structure.

In addition to the histamine binding pocket analyzed above, the second key interface for GPCR function is formed on the intracellular side between the GPCR and the α-subunit of a G protein. We analyzed the H_2_R-G_s_ interactions formed in our modeled complex and compared it to the interactions present in the β_2_AR-G_s_ template crystal structure used for modelling (Figure 4).

In both GPCRs the major interaction sites are formed by the intracellular loops (ICL1-3) and cytosolic ends of helices H3, H5, and H6. The key interacting residues within these regions (Figure 4) are frequently identical or at least exhibit conserved biophysical properties between H_2_R and β_2_AR. However, there are also differences in the G_s_ interaction pattern between H_2_R and β_2_AR in the loop connecting helix H7 and H8. This loop is one residue longer in H_2_R compared to β_2_AR and residues L291-R293 form a significantly larger number of contacts than the respective sequence patch in β_2_AR (Figure 4). This prompted us to compare the interaction of H_2_R and β_2_AR with G_s_ in more detail. The focus was on the polar interactions, which generally play an important role for the specificity of protein-protein recognition [47].

**Figure 5:**
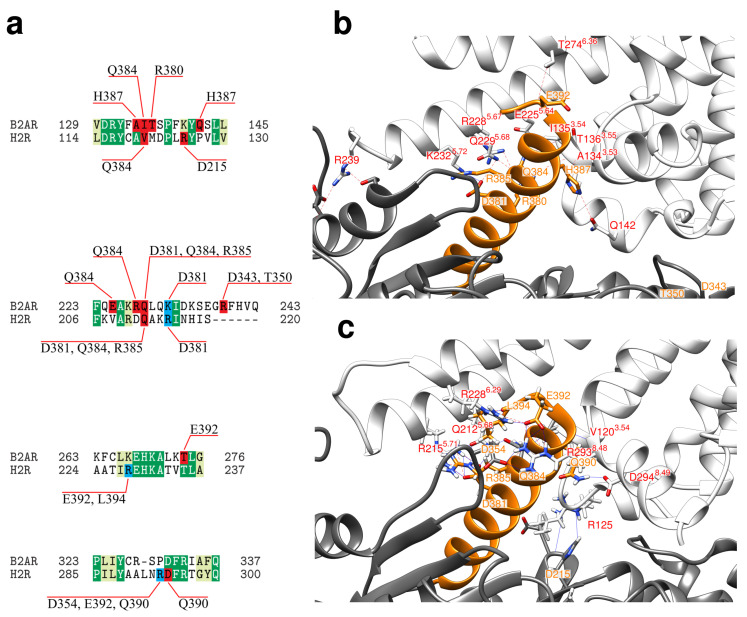
Specific interactions of the G_s_ protein in complex with H_2_R and β_2_AR. (**a**) Alignment of the sequence stretches from H_2_R and β_2_AR that form the main contacts with G_s_. Identical and similar residues are colored in dark and light green, respectively. Residues that form hydrogen bonds or salt-bridges to G_s_ are highlighted in red and blue, respectively. Interacting residues of G_s_ are given above and below the alignment. (**b**) Crystal structure of β_2_AR-G_s_ complex (white and gray ribbon) in complex with C-terminal helix G_s_-α5 highlighted in orange. Interface residues are shown in stick presentation and polar interactions are marked by dotted lines. Residues belonging β_2_AR and G_s_ are labeled in red and orange, respectively (**c**) Representative structure of H_2_R-G_s_ complex (white and gray ribbon) in complex with C-terminal helix G_s_-α5 highlighted in orange. Interface residues are shown in stick presentation and polar interactions are marked by dotted lines. Residues belonging to H_2_R and G_s_ are labeled in red and orange, respectively.

Figure 5 shows that both H_2_R and β_2_AR form numerous polar interactions with the C-terminal helix α5 of the G_s_-α-subunit. Both complexes contain a conserved salt bridge between D381 of the G_s_ protein and R215 in H_2_R, or K232 in β_2_AR. H_2_R exhibits two additional salt bridges formed by R228 and R293 in H_2_R, which have no structural equivalent in the β_2_AR complex (Figure 5a). R293 recognizes two acidic residues of G_s_–namely D354 and E392 (Figure 5c). R228 also recognizes E392 and in addition forms a salt bridge with the charged carboxy-terminus of L394 (Figure 5c). Taken together R228 and R293 of H_2_R are part of a comprehensive network of polar interactions that involves D354, E392, and L394 of G_s_. These contacts may offer an explanation for the observation that H_2_R interacts with G_s_ as a major signaling partner.

However, we want to emphasize that previous studies have shown that there is no simple GPCR sequence pattern explaining G protein coupling specificity [48,49] and that at least two additional structural factors may play an important role:

(i) Structural studies of GPCRs in complex with different G proteins indicate, that the position and dynamics of helix H6 significantly affect coupling selectivity [50,51,52].
(ii) As recently demonstrated for β_2_AR, transient interactions observed between the GPCR and GDP-bound G_s_ protein may represent an intermediate on the way to the formation of the final complex and may contribute to coupling specificity [53].

Future studies of H_2_R in complex with different G proteins or investigations of different G protein binding modes will be required to assess the role of these structural factors for H_2_R coupling preferences.

### 2.3. Conformational Stability of the Active H_2_R

One aim of the present study was to assess the role of intra- and extracellular binding partners in conformational stability of the active H_2_R. In this context, we compared the dynamics of the histamine-H_2_R-G_s_ ternary complex, the histamine-H_2_R binary complex, and apo H_2_R without any binding partner. In addition, a truncated ternary complex was generated that contains only the C-terminal G_s_-α5 helix (residues T369–L394) instead of the entire heterotrimeric G_s_ protein to investigate whether the α5 helix can sufficiently stabilize the active H_2_R. To enhance conformational sampling and facilitate large structural rearrangements, Gaussian accelerated MD (GaMD) simulations [23] were performed in addition to cMD simulations on all systems investigated (see Methods section for the simulation details).

**Figure 6:**
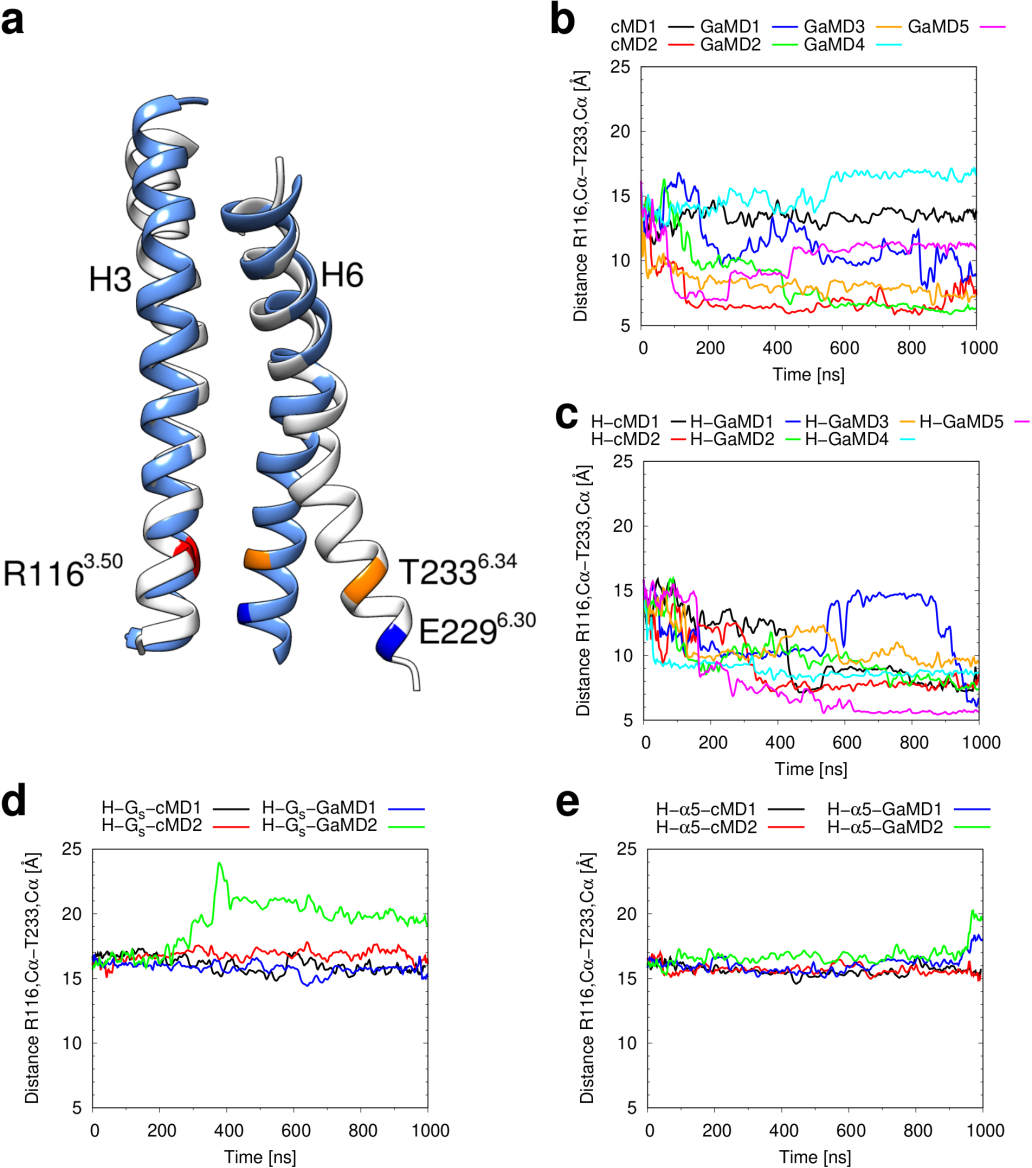
Inward motion of helix 6 in H_2_R. (**a**) Overlay of helices 3 and 6 from the active starting structure (white) and from a representative frame in which an approximation of the lower helix ends can be observed (light blue). For better orientation, R116^3.50^, E229^6.30^ and T233^6.34^ are marked in red, dark blue and orange, respectively. (**b**–**d**) Plots of the distance between the Cα atoms of the residues R116^3.50^ in helix 3 and T233^6.34^ in helix 6 as a function of simulation time for the simulations of (**b**) the apo H_2_R, (**c**) the H_2_R in complex with histamine, (**d**) the ternary complex of the H_2_R, histamine, and the G_s_ protein and (**e**) the complex of H_2_R, histamine, and the G_s_-α5 helix.

The most prominent motion detected in our simulations is the inward rotation of the intracellular end of helix 6 towards helix 3 (Figure 6a). This effect can be seen from the changes in the distance between the Cα atoms of R116^3.50^ and T233^6.34^ in simulations of the apo H_2_R (Figure 6b) and the H_2_R-histamine complex (Figure 6c). For the apo H_2_R, the motion may occur in less than 200 ns (see cMD2 and GaMD2 run), whereas in other simulations (cMD1, GaMD4) no approaching of the helices is observed on the timescales simulated (Figure 6b). This can be most likely attributed to the limited simulation time that does not allow a comprehensive sampling of slow motions, which are consequently only detected in a subset of the simulations. The simulations of the H_2_R-histamine complex (Figure 6c) qualitatively show the same behavior as the simulations of the apo H_2_R. In contrast, no decrease in H3–H6 distance is observed in the presence of G_s_ or G_s_-α5 (Figure 6d,e). This data suggests that histamine alone in unable to stabilize the active H_2_R, whereas addition of the G_s_ and G_s_-α5 can do so.

**Figure 7:**
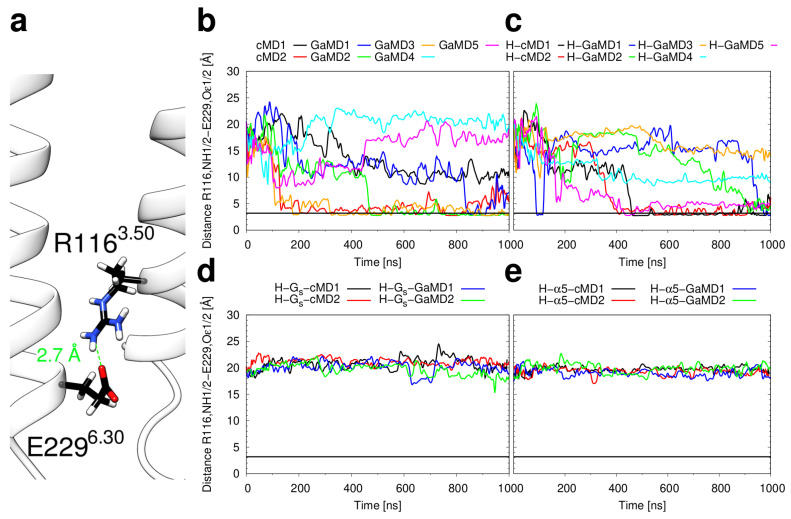
Formation of the ionic lock. (**a**) Representative frame from cMD1 showing the intracellular ends of helices H3 and H6 with the residues R116^3.50^ and E229^6.30^ displayed as sticks. Carbon atoms are shown in black, oxygen atoms in red, and nitrogen atoms in blue. (**b**–**e**) Plots of the minimum distance between any NH atom of R116^3.50^ and any Oε atom of E229^6.30^ as a function of simulation time for the simulations of (**b**) the apo H_2_R, (**c**) the H_2_R in complex with histamine, (**d**) the ternary complex of the H_2_R, histamine, and the G_s_ protein and (**e**) the complex of H_2_R, histamine, and the G_s_-α5 helix. The thick black horizontal line marks an N-O distance of 3.2 Å, which is the default cutoff criteria for a salt bridge in the MD visualization and analysis program VMD.

The inward motion of the intracellular end of H6 is generally described as a hallmark of GPCR inactivation [13,54]. We investigated whether additional structural features of inactive H_2_R emerge during our simulations. One such feature is the formation of the “ionic lock” between the oppositely charged sidechains of E229^6.30^ and R116^3.50^ (Figure 7). Ionic lock formation is observed in simulations of apo H_2_R and histamine-bound H_2_R (Figure 7b,c), but not in complexes containing the G_s_ or G_s_-α5 (Figure 7d,e). Comparison of the distances obtained from the individual simulations between Figure 6 and Figure 7 reveals that the inward motion of H6 generally correlates with ionic lock formation, i.e., the ionic lock appears to be the energetically most favorable sidechain arrangement when H3 and H6 are in close distance. This is remarkable, because the β_2_AR, which was used as a template for H_2_R modelling, does not display an ionic lock in the crystal structures of the inactive state (PDB code 5JQH [55]). However, in contrast to the crystal structure, long-timescale MD simulations of the β_2_AR show the ionic lock formation, which indicates the presence of a conformational equilibrium between conformations with the lock formed and the lock broken [56,57].

In summary, all analyses above indicate that the active conformation is retained in the ternary complex containing both histamine and the heterotrimeric G protein. In contrast, histamine alone is unable to stabilize this conformation. This is in line with previous work showing that the presence of the G protein is the key determinant for establishing an active conformation, whereas an agonist is insufficient to stabilize the active state [58,59]. In our study, the G_s_-α5 alone exerts a significant stabilization similar to that of the intact G protein. It is in line with the observation that mini G proteins or G protein mimicking nanobodies can stabilize the active conformation of GPCRs [33,59]. Microsecond cMD simulations have been able to capture beginning inactivation of the apo H_2_R and histamine-bound H_2_R in the absence the G_s_ or G_s_-α5. GaMD simulations yield mostly similar results on this aspect, while with certain improved sampling of larger conformational space in the receptor residue distances plotted in Figure 6 and Figure 7. Future GaMD simulations may be applied to investigate slower conformational changes underlying activation of the H_2_R and coupling of the receptor with different IBPs.

**Figure 8:**
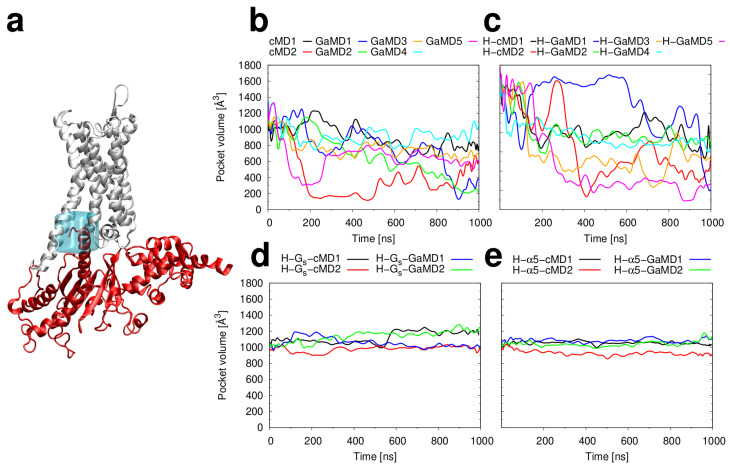
Volume of the G protein binding pocket. (**a**) Backbone representation of the H_2_R (white) and the G_s_ α subunit (red). The location of the G protein binding pocket is shown as a blue box. (**b**–**e**) Plots of the volume as a function of simulation time for the simulations of (**b**) the apo H_2_R, (**c**) the H_2_R in complex with histamine, (**d**) the ternary complex of the H_2_R, histamine, and the G_s_ protein and (**e**) the complex of H_2_R, histamine, and the G_s_-α5 helix.

The data above raises the question about the role of agonists for the stabilization of the active state. A recent study of $\beta_{1}$AR has shown that the presence of an agonist can shift the conformational equilibrium towards a pre-active state that facilitates G-protein binding [59]. In case of $\beta_{1}$AR, the conversion between these states occurs on a millisecond to second timescale [59] and can therefore not fully be covered by MD simulations. Despite the limited timescale accessible to MD simulations, there is some evidence from our simulations that the presence of histamine affects the conformational equilibrium-namely the volume of the G protein binding pocket compared to the apo H_2_R (Figure 8). In the presence of histamine, the volume of the pocket exhibits larger variations between the individual runs (Figure 8c), which might affect G protein binding.

Reciprocally, G protein coupling was reported to increase agonist binding affinity in case of the adenosine A_2A_ receptor between 10- and 40-fold [60,61]. For H_2_R, competitive binding experiments also indicate a   20-fold change of K_i_ in the presence of the G protein [62]. We have investigated this aspect in more detail by calculating the binding free energies for histamine in the presence and absence of G_s_ from the cMD simulations (Table 1). In the presence of G_s_, histamine is bound approximately 1 kcal·mol^−1^ tighter, which reflects the same trend as observed in previous experiments. Interestingly, this effect is not observed, when only the G_s_-α5 is present instead of the intact G protein (Table 1). This finding is a first clue that G_s_-α5, although it stabilizes the cytosolic part of H_2_R (Figure 6, Figure 7 and Figure 8), may be unable to enhance histamine binding affinity.

Taken together, the simulations of H_2_R in complex with different binding partners reveal that the presence of an intracellular partner (G_s_ or G_s_-α5) appears crucial for maintaining an active conformation, whereas histamine alone is insufficient to stabilize this state. These mechanistic properties are in good agreement with those described previously for the related $\beta_{1}$AR [59] and β_2_AR [58]. Our simulations also give first evidence for a weak allosteric coupling between the intra- and extracellular binding site in H_2_R, which has also been described for other GPCRs [13,63,64]. However, more and longer MD simulations or experiments with atomic resolution (NMR, X-Ray, cryo-EM) will be needed to obtain a comprehensive picture of the underlying molecular mechanisms.

## 3. Materials and Methods

### 3.1. Molecular Modeling of the H_2_R-G_s_ Complex

For model generation, the crystal structure of the human β_2_ adrenoceptor in complex with a G_s_ protein (PDB code 3SN6 [43]) was used as template. Both GPCRs exhibit a sequence identity of 31% (sequence similarity 66%). The homology model was generated with Modeller 9.16 [65] and comprises residues 15-304 of H_2_R. For the G_s_ protein, the sequence from PDB entry 3SN6 was kept with only one minor modification: Residues 60-87 of the α-subunit, which are not resolved in the template crystal structure, were replaced by a heptameric GSGSGSG-linker in the modeled complex. The camelid antibody fragment present in the template crystal structure was also kept in the modeled complex. The resulting model exhibited a good sterochemistry with 97% of the residues in the most favored regions of the Ramachandran plot and no steric clashes >0.35 Å.

### 3.2. Overview of the Molecular Dynamics Simulations

Preparation of the H_2_R-G_s_ complex including minimization, membrane embedding, and equilibration followed the protocol described for the H_1_R [66]. The parameters for histamine were also adapted from this work. An overview over all simulations performed is given in Table 2, which is subdivided in two sections: The first part contains information about the initial simulations that were used to derive the binding mode of histamine. The second part lists the simulations conducted for the final model to investigate H_2_R conformational stability.

Simulations to derive the binding mode of histamine were done with Gromacs [67]; the simulations conducted to investigate H_2_R conformational stability were done with AMBER [68]. The rationale for using two different simulation programs is that the PLUMED plugin required for metadynamics simulations works most efficiently with Gromacs, whereas the Gaussian-accelerated simulation method is not available for this program. The metadynamics protocol used for the determination of the histamine binding mode is described in detail in reference [66]. Briefly, Gromacs 2016.3 [67] was used in combination with the plumed 2.3.1 [69] plugin for all metadynamics simulations. The well-tempered metadynamics approach established previously by Saleh et al. [44], relies on the z component of the distances between the Cα atom of W247^6.48^ and the histamine ammonium nitrogen as collective variable (CV). The lower and upper walls $z_{\mathrm{low}}$ and $z_{\mathrm{up}}$ were chosen as 0.3 nm and 4.9 nm as CV boundary conditions. To discourage an irrelevant exploration of the bulk solvent the same bell-shaped funnel as described in [66] was used. The initial metadynamics simulation to capture the histamine binding pathway were run with an initial bias height of 7 kJ·mol^−1^, a gaussian width of 0.1 and bias factor 50. The multiple walker simulations were performed with bias factor 20 and an initial bias height of 5 kJ·mol^−1^.

### 3.3. Investigation of H_2_R Conformational Stability

The investigation of H_2_R conformational stability in the presence of different intra- and extracellular binding partners (Table 2) was performed with Amber [68] using the force fields ff14SB for the proteins, lipid14 for the DOPC molecules, and GAFF for histamine [70,71,72]. The initial structures were converted from the final structure of the cMD refined cluster5. Each system was energy minimized and equilibrated with the following protocol: Energy minimization consisted of three consecutive steps with restraints applied to different subsets of atoms (first to all atoms except for water molecules, second only to the Cα atoms, and third without any restraints). Each minimization stage was composed of 2500 steps with the steepest descent algorithm followed by 2500 steps of the conjugate gradient algorithm using a harmonic potential with a force constant of 10 kcal·mol${}^{-1}\cdot$Å${}^{-2}$ for the atom restraints. The equilibration was also performed in three consecutive parts which were conducted with a time step of 2 fs: First, the system was heated from an initial temperature of 10 K to 310 K within 0.1 ns while all atoms except for water molecules were restrained with a force constant of 5 kcal·mol${}^{-1}\cdot$Å${}^{-2}$. Keeping pressure and temperature constant (NPT ensemble), another 0.4 ns equilibration was performed where only the Cα atoms were fixed. Finally, the whole system was equilibrated for 0.5 ns without any restraints. The time step was 2 fs for all systems. The temperature was kept constantly at 310 K by a Berendsen thermostat [73]. A reference z pressure of 1 bar and a reference surface tension of 1.1 nm·bar were applied using surface-tension coupling. The SHAKE algorithm [74] was used during equilibration and production runs for bonds with hydrogen atoms. Periodic boundary conditions were set for x, y and z direction. The same general simulation parameters and equilibration strategies were used for both cMD and GaMD simulations. The GaMD protocol was inspired by a similar study for the muscarinic M2 receptor published by Miao et al. [33]. For the statistical calculation of the acceleration parameters, each GaMD run was preceded by a short 10.4 ns cMD simulation. This step was followed by a 32 ns equilibration in which the calculated boost potentials were added. For all GaMD simulations, the dual-boost mode was chosen which means that both the total potential energy and the dihedral energy terms were boosted. The reference energy was set to the lower bound ($E=V_{\max}$). Averages and standard deviations of the potential energies were updated every 400,000 steps (800 ps). For the standard deviations of the boost potentials applied to the total potential energy and the dihedral energy, an upper boundary of $\sigma_{0P}=\sigma_{0D}=6.0\;$kcal·mol${}^{-1}$ was defined. Table 3 gives an overview of the boost potentials (averages and standard deviations) that were actually added for the different simulation runs using these settings.

### 3.4. Structural Analysis

Post-processing and subsequent analysis were mainly performed using *cpptraj* from AmberTools 18 [68]. For the assessment of contacts, the *nativecontacts* command was used with a specified distance criteria of 5 Å. Interaction energies between ligand and receptor were calculated based on the MM/GBSA method using the script mm_pbsa.pl with default parameters [75,76,77].

The volume of the G protein binding pocket was calculated utilizing the tool POVME2 [78] according to a strategy published by Li et al. for the adenosine A_2A_ receptor [79]. The center of the binding pocket was defined as the geometric center of the last five Cα atoms of the α5 helix of Gsα. In case of simulations without the G_s_ protein, an overlay with a G_s_-bound H_2_ receptor was performed to determine the respective coordinates. A 12 × 12 × 12 Å${}^{3}$ rectangular box was defined as the maximum extension of the binding pocket. PISA [80] was used for interface analysis. Structure visualization was done with UCSF Chimera [81] and VMD [82] while plots were created with gnuplot [83]. For the visualization of sequence alignments, the shade package [84] was used.

## 4. Conclusions

The H_2_R represents an established drug target for the treatment of gastric diseases [8,9] and is also actively studied as a target for reducing the risk of heart failure [11]. Since there is yet no experimental structure available for H_2_R, we have generated a computational model of the ternary histamine-H_2_R-G_s_ complex and assessed its conformational dynamics. Ligand binding and conformational changes of GPCRs mostly occur on timescales longer than microseconds [13,14] and can be covered only in part by cMD simulations. Therefore, metadynamics and GaMD simulations have been applied to enhance conformational sampling.

A multiple walker metadynamics simulation was used to identify the histamine binding mode, which was subsequently refined by cMD simulations according to an existing protocol [41]. The resulting histamine binding mode was in good agreement with data from site-directed mutagenesis [46] underscoring the usefulness of a cMD/metadynamics combination for the determination of GPCR ligand binding modes.

The analysis of the H_2_R-G_s_ interface revealed a comprehensive network of polar interactions supporting the H_2_R preference for G_s_ as a major signaling partner. However, there are additional structural factors that affect G protein coupling specificity, e.g. the exact position of helix H6 [50,51,52] or the existence of structural intermediates on the binding pathway [53]. Therefore, future studies of H_2_R in complex with different G proteins or investigations of different G protein binding modes will be required to assess the role of these structural factors for H_2_R coupling preferences.

We also compared dynamics of the histamine-H_2_R-G_s_ ternary complex, the histamine-H_2_R binary complex and the apo H_2_R to assess the role of different interaction partners in the conformational stability of the active H_2_R. In addition, a truncated ternary complex that contains only the C-terminal G_s_-α5 helix instead of the entire G_s_ protein was simulated to investigate whether the α5 helix could sufficiently stabilize the active state. To enhance conformational sampling and facilitate large structural rearrangements, 1-μs GaMD simulations were performed in addition to cMD simulations.

During the simulations of the apo H_2_R and the H_2_R-histamine complex, several structural features typical for inactive GPCRs emerged: An inward motion of the intracellular end of helix H6, the formation of the “ionic lock”, and a contraction of the G protein binding pocket. In contrast, these motions were not observed in the presence of G_s_ or G_s_-α5, suggesting that the presence of an intracellular partner is crucial for maintaining an active GPCR conformation, whereas histamine alone is insufficient to stabilize this state. This was in line with previous studies showing that the G protein is the key determinant for generating an active conformation, whereas an agonist alone is insufficient to stabilize the active state [58,59]. In addition, the simulations also provided first indications for a weak allosteric coupling between the intra- and extracellular binding sites, which has also been described for other GPCRs [13,63,64]. However, our investigations also showed that certain motions in H_2_R are so slow that they cannot be fully sampled even using 1-μs GaMD simulations. Therefore, future cMD and GaMD simulations with longer simulation times or modified simulation parameters will be required to investigate slower conformational changes underlying H_2_R activation.

## Figures and Tables

**Table 1:** Binding energies between histamine and the H_2_ receptor. Analysis were performed using cMD simulations of the binary histamine-H_2_R complex (H-cMD1/2), the ternary histamine-H_2_R-G_s_ complex (H-G_s_-cMD1/2), and the ternary complex including only G_s_-α5 instead of G_s_ (H-α5-cMD1/2). Averages and standard errors of mean (*n* = 10,000 frames from the second half of the simulations) were calculated for the binding energies using MM/GBSA.

System	Binding Energy [kcal/mol]
H-cMD1	–13.13 ± 0.02
H-cMD2	–13.09 ± 0.04
H-G_s_-cMD1	–14.36 ± 0.03
H-G_s_-cMD2	–13.87 ± 0.04
H-α5-cMD1	–13.41 ± 0.03
H-α5-cMD2	–11.76 ± 0.04

**Table 2:** Overview of the simulations performed. The table lists all MD simulations conducted for the present study and the respective names by which they are referred to in the figures and manuscript text. It is stated whether a simulation was a conventional MD simulation (cMD), a Gaussian accelerated MD simulation (GaMD), or a metadynamics simulation. The number of runs and the respective simulation times are listed. Furthermore, the table describes the composition of the respective systems, i.e., the box dimensions and whether the histamine H_2_ receptor (H_2_R), the ligand histamine (HSM), the G_s_ protein (✓), or the G_s_-α5 helix (⋋) was present. The symbol (×) denotes the absence of the respective component in the setup.

Simulation Name	Runs × Time	H_2_R	HSM	G_s_	#Atoms	#Water	#DOPC	Water Box Dimensions
Deduction of histamine (HSM) binding mode								
Multiple walker metadynamics	32 × 48 ns	✓	✓	✓	355,534	82,961	638	14.5 Å × 14.5 Å × 16.5 Å
Refinement cMD cluster{1–5}	5 × 1 μs	✓	✓	✓	355,534	82,961	638	14.5 Å × 14.5 Å × 16.5 Å
Validation of stability and switchability								
cMD{1–2} GaMD{1–5}	7 × 1 μs	✓	×	×	125,620	27,519	278	9.6 Å × 9.7 Å × 13.0 Å
H-cMD{1–2} H-GaMD{1–5}	7 × 1 μs	✓	✓	×	125,638	27,519	278	9.6 Å × 9.7 Å × 13.0 Å
H-G_s_-cMD{1–2} H-G_s_-GaMD{1–2}	4 × 1 μs	✓	✓	✓	355,534	82,961	638	14.5 Å × 14.5 Å × 16.5 Å
H-α5-cMD{1–2} H-α5-GaMD{1–2}	4 × 1 μs	✓	✓	⋋	342,067	110,592	638	14.5 Å × 14.5 Å × 16.5 Å

**Table 3:** Boost potentials of the Gaussian accelerated molecular dynamics (GaMD) simulations. All GaMD simulations were run with the dual boost option which means that both the total potential energy and the dihedral energy were boosted. Averages ± standard deviations of the potentials applied are listed for the different simulations.

Simulation Name	Total E${}_{\mathrm{pot}}$ Boost [kcal/mol]	Dihedral Energy Boost [kcal/mol]
GaMD1	7.19 ± 3.11	6.61 ± 2.67
GaMD2	7.17 ± 3.06	6.56 ± 2.67
GaMD3	7.47 ± 3.16	6.72 ± 2.70
GaMD4	7.70 ± 3.19	6.63 ± 2.69
GaMD5	7.64 ± 3.16	6.05 ± 2.55
H-GaMD1	8.52 ± 3.48	6.24 ± 2.59
H-GaMD2	7.59 ± 3.18	5.94 ± 2.55
H-GaMD3	7.45 ± 3.14	6.26 ± 2.61
H-GaMD4	7.56 ± 3.22	6.16 ± 2.57
H-GaMD5	7.27 ± 3.10	6.15 ± 2.57
H-G_s_-GaMD1	34.72 ± 6.85	6.68 ± 2.76
H-G_s_-GaMD2	7.89 ± 3.56	7.51 ± 2.94
H-α5-GaMD1	10.21 ± 4.39	6.68 ± 2.73
H-α5-GaMD2	7.81 ± 4.00	6.87 ± 2.79
